# Dyslipidemia After Kidney Transplantation and Correlation With Cyclosporine Level: A Glimpse into the Future

**DOI:** 10.5812/numonthly.14179

**Published:** 2013-11-13

**Authors:** Mohamed H. Ahmed

**Affiliations:** 1 Department of Medicine, Milton Keynes Hospital NHS Foundation Trust, Milton Keynes, UK

**Keywords:** Lipids, Kidney Transplantation, Cyclosporine

***Dear Editor,***

I read with interest the article by Hosseini et al. recently published in the Nephro-Urology Monthly, titled “dyslipidemia after kidney transplantation and correlation with cyclosporine level”. They concluded that dyslipidemia is common finding after kidney transplantation and has no correlation with Cyclosporine level (1). Importantly, serum creatinine was the only risk factor for hypercholesterolemia development after kidney transplantation. Furthermore, in this study hypercholesterolemia, hypertriglyceridemia and low high density lipoprotein (HDL-C) were observed more in the cadaveric kidney transplantation. This may suggest the ischemia-reperfusion injury during organ retrieval and transplantation may have a role in the development of dyslipidaemia post transplantation. Despite the fact that 75% of total population in Hosseini et al. study received living transplant, they noticed cholesterol and triglyceride levels were significantly higher 4 to 12 months after transplant than their level 3 months and beyond 1 year after renal transplantation. Interestingly, Tse et al. have shown that incidence of dyslipidemia decreased over the time after kidney transplantation (2). Generally speaking, in renal transplant patients, the pattern of dyslipidemia is characterized by elevated plasma levels of total cholesterol (TC), low density lipoprotein (LDL-C), very low density lipoprotein (VLDL-C), and high triglyceride (TG) in addition to markedly reduced high density lipoprotein (HDL-C) (3, 4). It is worth mentioning, that there is no clear pattern of dyslipidemia associated with renal transplantation and currently it is difficult to conclude whether dyslipidemia will worsen or improve over the time after kidney transplantation. This can be attributed to the fact that dyslipidemia after renal transplant are usually multifactorial. The renal transplant patients have increased risk of cardiovascular disease due to increased prevalence of hypertension, dyslipidemia and diabetes (5). Other non-traditional risk factors include, hypothyroidism, excessive alcohol consumption, chronic liver disease, left ventricular hypertrophy, cardiomyopathy, proteinuria, medications induced dyslipedemia, and uremic toxins (5). The unintended effects of immunosuppression (diabetes, hypertension, uremia and anemia) are also important. Therefore, careful assessment of dyslipidemia after renal transplantations is prudent. Furthermore, Tacrolimus, Azathioprine and Mycophenolate mofetil usually induce only minor changes in serum lipid profile. It has been shown that conversion of cyclosporine to one of these drugs is followed by significant decrease in the levels of total and LDL-c (6, 7). The use of lipid lowering medication as treatment for dyslipidemia post-renal transplantation have attracted a lot of research within the last decade and most of the related clinical trials were conducted in the West. Indeed, further clinical trials in the region of Middle East and Persian Gulf may reveal an exciting outcome as genetic predisposition is another factor leading to post transplant dyslipidemia.
